# Telehealth coaching in older adults, behavior change, and impacts of the COVID-19 pandemic: analyses from The Brain Health Champion Study

**DOI:** 10.3389/fdgth.2025.1510804

**Published:** 2025-04-17

**Authors:** Brittany McFeeley, Casey Nicastri, Taylor Krivanek, Kirk R. Daffner, Seth A. Gale

**Affiliations:** ^1^ Brigham and Women’s Hospital, Department of Neurology, Boston, MA, United States; ^2^ Harvard Medical School, Boston, MA, United States

**Keywords:** behavior change interventions, health coaching, COVID-19, older adults, MCI (mild cognitive impairment), Alzheimer's disease and related dementias (ADRDs), brain health, digital/mobile health

## Abstract

**Introduction:**

When COVID-19 containment strategies were imposed in March 2020, we became interested in how these restrictions might interfere with brain-healthy behaviors of older adults who were either actively participating in or who had recently completed our telehealth behavior change intervention. Telehealth interventions have emerged as important tools for supporting brain health behaviors remotely, particularly among older adults. The objective of the current study was to assess how older adults with and without cognitive impairment were affected by COVID-19 restrictions and whether they were affected differently based on their active participation or recent completion of our Brain Health Champion (BHC) study and their cognitive status.

**Methods:**

BHC study 1.0 and 2.0 participants and their study partners were emailed in April and May of 2020 a link to five electronic surveys to collect qualitative and quantitative data on various health factors, including self-reports of pre-pandemic and current brain health behaviors (e.g., physical activity, Mediterranean diet adherence, social engagement, and cognitive stimulation), anxiety, sleep, and depression. The fifth survey was distributed to collect study feedback.

**Results:**

Ten out of 11 participants from Study 2.0 and 15 out of 30 participants from Study 1.0 completed the surveys. Results demonstrated that early pandemic restrictions negatively impacted all participants in physical activity (*p* < .01) and social interactions (*p* < .001), with no impact on cognitive activities (*p* = .479) and dietary intake (*p* = .814). A significant difference was found between Study 1.0 and 2.0 participants (*p* < .001) in self-reported changes in level of cognitive activity. Study 1.0 participants indicated a decrease in cognitive activities since the start of COVID-19 restrictions, whereas those in Study 2.0 reported an increase in cognitive activities.

**Discussion:**

Our findings suggest that pandemic restrictions significantly impacted activities typically done outside the home (social and physical activity), while those feasibly achieved at home were less affected (Mediterranean diet adherence and cognitive activity). Additionally, the intervention augmented by digital health components likely exerted some protective effects against the impact of COVID-19 containment strategies. Digitally-facilitated research and clinical telehealth programs are well-positioned to offer some protection to vulnerable individuals from disruptive events that could impede adoption or maintenance of healthy lifestyle changes.

## Introduction

The World Health Organization (WHO) officially declared coronavirus disease 2019 (COVID-19) a global pandemic on March 11, 2020. Countries across the world quickly began to implement containment strategies to slow the spread of the virus, including physical distancing and mask mandates, non-essential business closures, and curfews (1). These recommendations and mandates issued by local governments or organizations, like U.S. Center of Disease Control (CDC) and its world-wide equivalents, confined many individuals largely to their homes, except when required to meet essential needs, like obtaining food or emergency medical care. Shortly after the start of the pandemic, there was abundant discussion in the media that the deleterious effects COVID-19 restrictions were likely to have on overall health and wellbeing; soon after, health researchers began to report on these emerging negative effects (2, 3).

Several studies published their findings on the change in health behaviors due to these COVID-19 containment strategies. A study by Weaver and colleagues surveyed a representative sample of adults (18+) in several regions of the U.S. in April and May of 2020, near the beginning of widespread COVID-19 containment strategies, to assess changes in health behaviors before and during their implementation. Substantial decreases in physical activity and social stimulation were found after the COVID-19 restrictions were imposed compared to pre-pandemic levels, while no change in diet quality was found (2). It has been well-demonstrated that routine physical activity and social stimulation, among other modifiable risk factors for dementia and cognitive decline, are important for maintaining cognitive health (4–6). The evidence from the Weaver et al. study and others suggests that the COVID-19 pandemic containment strategies adversely influenced individuals' practice of brain healthy behaviors and had negative health consequences across populations which may have longer-term health implications (2, 7). A study by Dor-Haim et al. found that COVID-19 containment strategies were related to an increase in sedentary behavior in active adults and, consequently, weight gain (8). Additionally, the pandemic caused significant hindrances to clinical research participation, particularly in studies that involve behavioral interventions (9, 10).

Many behavioral interventions with digital health components started prior to the pandemic and some continue to investigate the impact of physical activity and adherence to a Mediterranean diet on various health outcomes, such as cognitive aging (11–13), cardiovascular health (14), mental health (15, 16), and diabetes (17). Other behavioral interventions have investigated the influence of social and cognitive engagement on cognitive aging (11, 18–20). Both ongoing and completed lifestyle interventions may have been disrupted by the pandemic's restrictions by reducing or ceasing the enrollment of new participants, hindering participants' progress in active studies, and interfering with the maintenance of behaviors adopted following completed studies.

The adoption of healthy lifestyle behaviors is urgently needed to reduce global dementia risk, with 6.9 million people in the United States alone affected by just Alzheimer disease (AD). By 2060, that number is projected to reach 13.8 million (21, 22). In 2020, COVID-19 contributed to a 17% increase in deaths among patients diagnosed with Alzheimer's and related dementias (21). During March of 2020, we were actively recruiting older individuals with mild cognitive impairment or risk factors for dementia for a behavioral, mobile health-augmented intervention called the Brain Health Champion (BHC) study. This was the second cohort of participants in BHC, called “Study 2.0”, with a total of 11 participants at the start of the pandemic (8 active; 3 completers). This cohort followed the first BHC study cohort, “Study 1.0” (*N*=∼40), which was completed in 2018. Both BHC Study 1.0 and Study 2.0 were six-month interventions investigating the impact of health coaching in augmenting routine primary and neurological care, compared to routine care alone, to promote and maintain brain healthy, dementia risk-reducing behaviors. In Study 1.0 and Study 2.0, participants were randomized to either the health coach intervention group or the Physician Counseling and Education (PCE) group, an active control, which consisted of routine neurologic/primary care, plus distribution of education materials on brain health by email and/or regular mail every six weeks throughout the six-month program.

Study 1.0's intervention involved a health coach who delivered a 15-min phone-based coaching session each week and face-to-face visit every six weeks, to participants with subjective cognitive disorder (SCD), mild cognitive impairment (MCI), or mild dementia (MD) due to AD. The weekly phone calls were designed to motivate participants to increase their physical activity engagement, to eat healthier, and to engage in cognitively and socially stimulating activities. The content of conversations and short-term goal setting were unique to each participant, in order to create a personalized health coaching experience. Health coaches tracked progress and adjusted goals accordingly. Details of the methods can be found in the original article (12). The ongoing Study 2.0's intervention advances the weekly visits of 1.0 by using a smartphone-based mobile health platform, Fruit Street Health (23), to facilitate weekly face-to-face video calls, text messaging, and a one-time consultation with a dietitian to both participants at-risk for AD and those with a diagnosis of mild cognitive impairment (MCI).

Study 1.0 demonstrated clinically and statistically significant effects compared to routine care of increased adherence to brain-healthy behaviors during its six-month phone intervention, with face-to-face visits every six weeks, and improved self-reported quality of life in participants with varying degrees of cognitive impairment (12). Based on preliminary analysis of Study 2.0 data, the health coach plus mobile health intervention also seems to promote adherence to a Mediterranean diet, increased cognitive activity, lower depression scores, and higher quality of life (24).

When pandemic restrictions were implemented, we paused recruitment for Study 2.0 for several weeks and assessed how to revise the recruitment process and study procedures to allow the study to continue. Ultimately, we converted all six-week visits (including the dietitian visit) and study procedures to use our virtual, mobile health platform. Given the evidence of the pandemic's negative impact on mental health and the practice of healthy behaviors, we were interested in whether COVID-19 restrictions that involved disruption of daily routines were stymying progress in the maintenance of adopted lifestyle behaviors and having an adverse impact on mood, perceived stress, and sleep in our study population. Specifically, we were interested in how participants who completed our BHC study would be affected by COVID-19 confinement strategies and whether these strategies seemed to directly impact individuals' participation in learned and adopted behaviors.

The goal of the current study was to assess how participants, who either completed or were actively participating in a brain health behavioral intervention that promoted behaviors such as physical exercise and social stimulation, were affected by COVID-19 confinement strategies. We hypothesized that the pandemic would be tied to a decrease in current levels of brain-healthy behaviors adopted and being practiced by individuals who completed both BHC study cohorts (1.0 and 2.0) or were still active in 2.0, with lesser change in those who completed the health coaching intervention (active arm) than who received only provider counseling and education (PCE active control arm). Due to the ease of connection by telephone or digital platform, the actual work with their health coach, and the offer of that coach relationship of an extrinsic, social influence that might persist through a major disruption in daily activities, we predicted that participants randomized to health coaching might be more resistant to negative influences of the pandemic on brain healthy behaviors than their counterparts in usual care.

## Materials and methods

### Recruitment

Brain Health Champion Study 1.0 and 2.0 completers and those active in 2.0, along with their study partners (only for participants who were diagnosed with MCI or mild dementia) were notified by email and/or letter in April 2020 of the study team's interest in collecting additional information via surveys to better understand how the COVID-19 pandemic was impacting them and impacting benefits potentially accrued or accruing from their study participation. Participants were sent another email and/or electronic message through the Mass General Brigham (MGB) Electronic Medical Record (EMR) patient portal, Patient Gateway, including a hyperlink to our electronic surveys. Additional reminder phone calls to participate were made in June 2020 to participants and study partners who had yet to complete the surveys. Study data were collected and managed using Research Electronic Data Capture (REDCap) electronic data capture tools accessed through MGB (25, 26). REDCap is a secure, web-based software platform designed to support data capture for research studies, providing (1) an intuitive interface for validated data capture; (2) audit trails for tracking data manipulation and export procedures; (3) automated export procedures for seamless data downloads to common statistical packages; and (4) procedures for data integration and interoperability with external sources (25, 26). If participants could not complete the surveys electronically, we sent paper copies via USPS mail.

### Participants

The only inclusion criterion for the current study was that an individual had participated in BHC Study 1.0 or 2.0 (*N* = 55). Study 1.0 participants were all older individuals with either MCI or mild dementia, and 2.0 participants had either MCI mild cognitive impairment or were cognitively normal with risk factors for dementia. Subjects from Study 1.0 (*n* = 8) were excluded if they had become too cognitively impaired to respond to the questionnaires provided. Six participants from Study 1.0 had died since completing their intervention, four who were in the BHC arm and two in the PCE control arm. The online surveys were distributed to all living study completers from Study 1.0 (*n* = 38) and active participants and completers of Study 2.0 (*n* = 11); In a small number of other cases, study partners assisted participants from Study 1.0 with completion of the surveys due to participants' cognitive decline.

### Measures

There were five online surveys designed to collect quantitative and qualitative data on various health factors in addition to general feedback on the BHC studies. The main survey addressed participants' current vs. pre-pandemic participation in brain health behaviors (labelled “Main BHC Survey”), a second set of surveys addressed their current neuropsychiatric status (i.e., anxiety, sleep, and depression; labelled “Additional Health Questionnaires”), and a third survey (labelled “Feedback Survey”) asked for participants' overall feedback on the study and its interventions.

#### Main BHC survey

The Main BHC Survey had 12 questions which consisted of multiple choice and five-point, Likert-type scales, with some questions followed by open-ended prompts allowing respondents to elaborate on responses. Questions were developed by all authors to understand changes in physical activity, dietary habits, cognitive stimulation, and social activity since the start of the pandemic, presumably due to implementation of containment strategies. For example: “*How does your current level of physical activity compare to your activity prior to March 2020?*” Responses to this question included “Significantly less; Somewhat less; About the same; Somewhat more; Significantly more.” Immediately after this question there was a follow-up prompt which stated: “*Please explain how your physical activity has or has not changed due to the pandemic*.” This question structure remained consistent for all other outcome measures, including dietary habits, cognitive activity levels, and social engagement. (See Supplementary Material S.1 for the complete “Main BHC Survey”).

#### Additional health questionnaires: sleep quality

Sleep quality was measured using the Sleep Quality Scale (SQS). The SQS is a 28-item scale designed to measure six domains of sleep quality: daytime symptoms, restoration after sleep, difficulty waking, and sleep satisfaction (27). Using a four-point, Likert-type scale, participants indicated how frequently they experience certain sleep behaviors (0 = rarely, 1 = sometimes, 2 = often, and 3 = almost always). Some of the items include: “I have difficulty falling asleep”; “I wake up while sleeping”; “Poor sleep gives me headaches”; and “I feel refreshed after sleep”. Scores on the SQS range from 0 to 84, in which higher scores represent greater problems with sleep. Items capturing “restoration after sleep” and “satisfaction with sleep” were reverse coded before being tallied. The internal consistency of the test is.92 and its test-retest reliability is.81 (27).

#### Additional health questionnaires: anxiety

Anxiety was measured using the General Anxiety Disorder screener (GAD-7). The GAD-7 screener is a reliable tool for identifying the presence and probable cases of Generalized Anxiety Disorder. The GAD-7 consists of 7-items in which participants indicated how frequently they have been bothered with the following problems: “feeling nervous, anxious or on edge”; “not being able to stop or control worrying”; “worrying too much about different things”; “trouble relaxing”; “being so restless that it is hard to sit still”; “becoming easily annoyed or irritable”; and “feeling afraid as if something awful might happen.” Reponses were coded using a four-point, Likert-type scale ranging from 0 (not at all) to 3 (nearly every day). Each of the seven items were added together and higher scores indicated higher anxiety symptom severity (scores ranging from 0 to 21). (28) This scale has been previously used in older adult populations (29, 30).

#### Additional health questionnaires: depression

Depression was measured using the Patient Health Questionnaire (PHQ-9). The GAD-7 anxiety screener is typically administered along with PHQ-9 depression screener when assessing both anxiety and depression symptoms. The PHQ-9 questionnaire is a 9-item survey that was designed to assess depression symptom severity and how frequently they experience the symptoms. Some of the items include: “little interest or pleasure in doing things”; “feeling down, depressed, or hopeless”; “trouble falling or staying asleep, or sleeping too much”; and “feeling tired or having little energy.” Reponses were coded using a four-point, Likert-type scale ranging from 0 (not at all) to 3 (nearly every day). Each of the nine items were summed together, and higher scores mark more severe depressive symptoms (scores ranging from 0 to 27). (31) The PHQ-9 has been also validated in neurologic populations (32).

#### Feedback survey

The Feedback Survey was used to gather some qualitative data. It was created to capture whether participants felt that their participation in the study (past or present) had any impact on stressors caused by the pandemic and if so, which aspects of the study were helpful. We asked participants the following question: “Do you think your involvement in the Brain Health Champion study has had any impact on your response to additional personal stresses (e.g., feeling lonely or isolated, not being able to see loved ones or close friends in-person, having medical appointments postponed) that were brought on by the pandemic?” Responses included yes or no. If participants answered yes, then we asked them to rate impact [(1): Positive impact; (2): Neutral impact; (3): Negative impact] and another follow-up question: “What aspects of the study, if any, have had an impact on your coping response to the pandemic and/or your quality of life during this time?” Participants were asked to check as many of the following that applied: “knowing you are being encouraged by a health care team to adopt behaviors that promote health”; “knowing that you are connected through a weekly video call and possible text messaging to the health coach”; “knowing that you are involved in a medical research study of any kind”; “knowing that you are involved in a medical research study that is specifically trying to advance our understanding of the brain and wellness”; “your relationship with your health coach in this study”; and “the educational materials about brain health that have been distributed to you as part of the study.” Participants were then asked, “please describe any other thoughts, feelings, or personal reactions that you have about the COVID-19 pandemic and/or the Brain Health Champion Study.” Responses were coded into six themes: personal concerns, concerns for others, optimism for the future, grateful for participation, experienced negative behavior change, and experienced positive behavior change. Responses could have more than one theme.

#### Statistical approach

We performed descriptive analyses for our study sample as a whole and for each diagnostic group (e.g., SCD/at risk, MCI, and dementia) separately. One-sample *t-*tests were conducted on the four main outcomes across all participants of the BHC COVID-19 sub-study: change in (1) physical activity, (2) diet, (3) cognitive activity, and 4) social engagement from the start of the pandemic to the time that the surveys were distributed to determine if the change measured was significantly different from their behaviors pre-pandemic. An independent samples, two-tailed *t*-test, with unequal variances was used to analyze differences in the four main outcomes between intervention-type (phone/mobile health coach vs. PCE) in change of the four brain health behaviors. An independent samples, two-tailed *t*-test, with unequal variances was used to analyze differences between intervention-type (phone/mobile health coach vs. PCE) in the four main outcomes variables. To test for the change across diagnostic groups (at-risk/SCD, MCI, and dementia), a one-way analysis of variance (ANOVA) test of between-subject factors with *post hoc* tests (using Bonferroni correction to adjust *p*-values due to unequal variances and unequal group sizes) was used to determine any significant differences across our four main outcomes. A one-way ANOVA test was also used to determine whether there were significant differences between diagnostic groups for sleep quality, anxiety, and depression. Qualitative results were reported descriptively, and the responses to the open-ended question were thematically coded by two authors. Authors coded themes separately and double-checked coding for inter-rater reliability. Any discrepancies in coding were discussed and re-analyzed to reach an agreement. All statistical analyses were performed using SPSS version 27. Tests with *p*-values of <.05 were considered statistically significant. Our sample size was too small to perform a meaningful power calculation.

## Results

We had a 61% response rate of the 41 possible participants. Fifteen participants from Study 1.0 and ten participants from Study 2.0 (*N* = 25), including those at risk/subjective cognitive decline (SCD) (*n* = 7), with MCI (*n* = 13), and with mild dementia (*n* = 5), attempted to complete the surveys. Two participants from Study 1.0, both with a diagnosis of dementia, only partially completed the surveys; one completed only the “Main BHC Survey,” while the other completed only the “Additional Health Questionnaires.” Thus, 24 participants were included in the “Main BHC survey” and “Additional Health Questionnaires” analyses. While there were notable and statistically significant differences found in our analyses described below, conclusive interpretation of results is limited by our small sample size and inherent limitations of the brief screening tools used.

### Main BHC survey results

**Figure 1:**
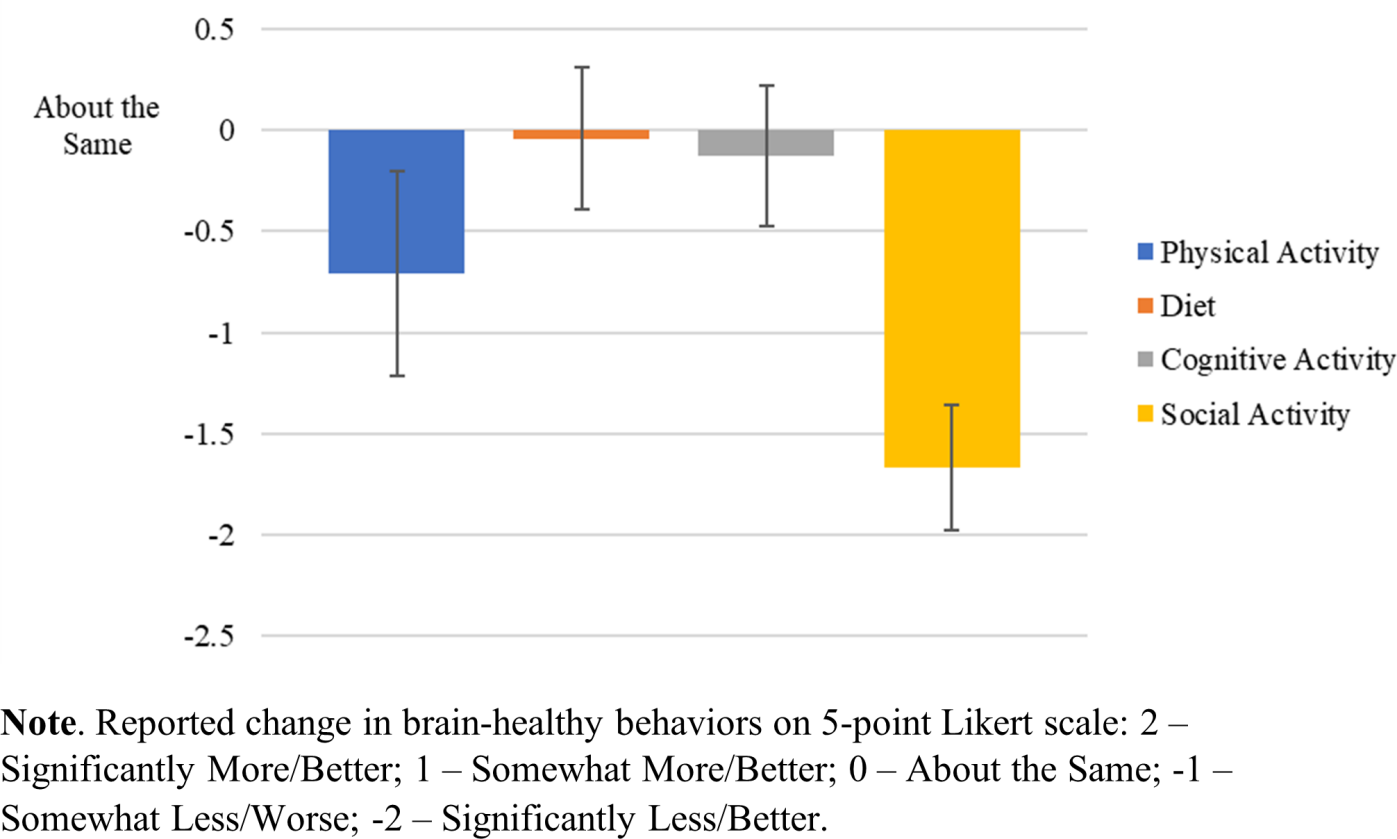
Impact of COVID-19 on brain-healthy behaviors on all surveyed participants.

Table 1 provides demographic information for all respondents from both BHC Study 1.0 and Study 2.0 (*N* = 25). For simplicity, participants with a diagnosis of SCD and participants recruited based on their at-risk status, were grouped together for analysis due to their similar levels of cognitive functioning. The response rate for full or partial completion of the e-surveys was 61% (25/39), with 24/25 fully completing the “Main BHC Survey” and 24/25 fully completing the “Additional Health Questionnaires.” Social engagement was markedly disrupted [*t*(23) = −10.724, *p* < .001] on average across surveyed participants in both study cohorts (1.0 and 2.0). Physical activity was moderately undermined [*t*(23) = −2.815, *p* < .01]. Cognitive activity was not significantly affected [*t*(23) = −.72, *p =* .479], nor was diet, with little to no change before or after pandemic-related restrictions [*t*(23) = −.238, *p* = .814]. See Figure 1.

**Table 1:** Demographic characteristics (*N* = 25).

	SCD/at-risk (*n* = 7)	MCI (*n* = 13)	Mild dementia (*n* = 5)
Age (*M*, years)	65.6	71.5	72.6
Sex (Male/Female)	3/4	7/6	5/0
Ethnicity (Non-Hispanic/Hispanic)	6/1	13/0	5/0
Proportion in BHC arm	.57	.30	.40

Note. Diagnoses represent participant diagnosis at their enrollment into the BHC study.

SCD, subjective cognitive decline; MCI, mild cognitive impairment; BHC, Brain Health Champion.

All participants are White.

**Figure 2:**
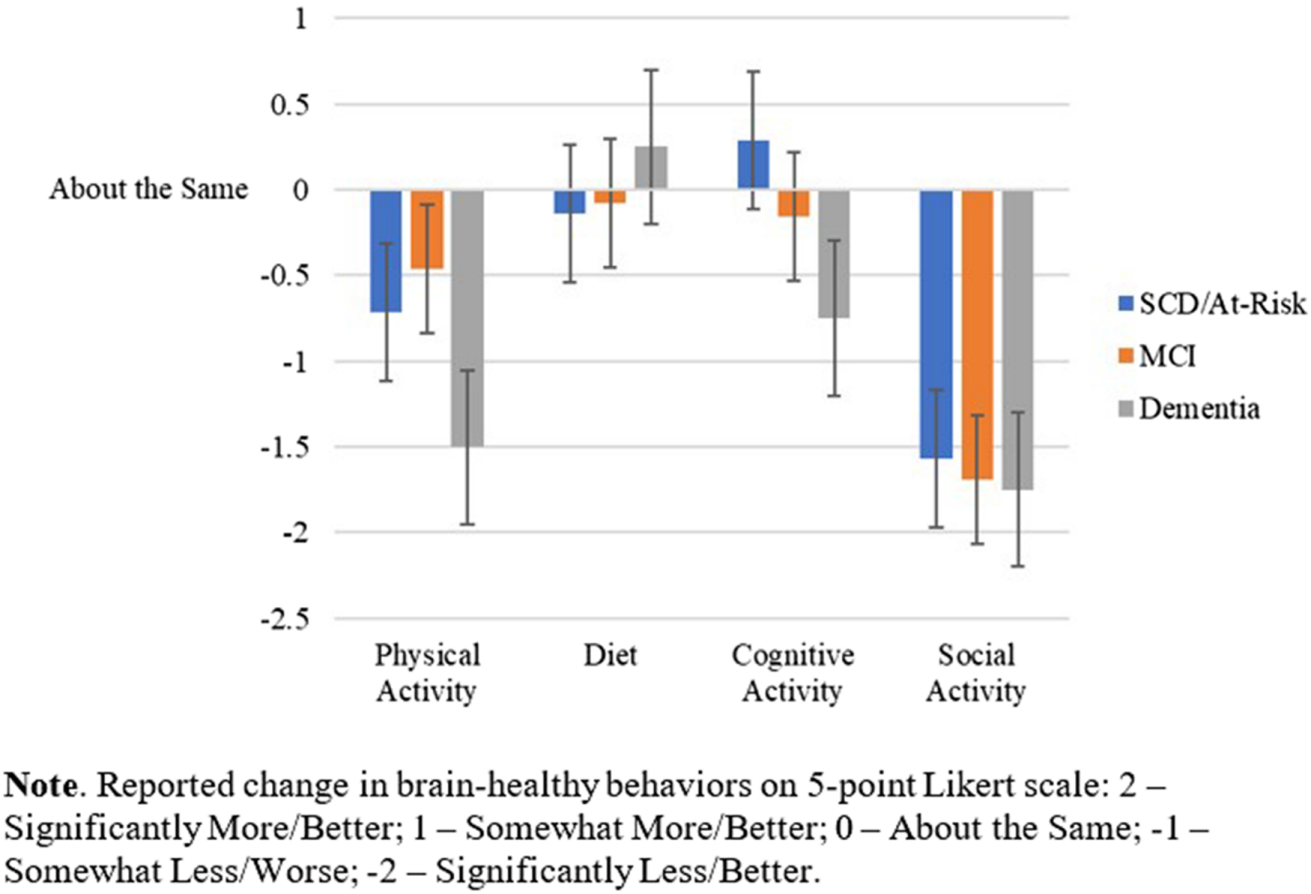
Impact of pandemic confinement strategies on brain-healthy behaviors across diagnostic groups.

Table 2 shows the comparison of the four main outcomes across the intervention type (phone/mobile health coach vs. PCE-active control). Intervention type did not influence the impact of COVID-19 on these brain healthy behaviors. Table 3 shows the comparison of the four main outcomes by study participation (Study 1.0 vs. Study 2.0). A statistically significant difference was found in self-reported changes in level of cognitive activity (*p* < .001) between studies. Individuals in Study 1.0 indicated a decrease in participation in cognitive activities since the start of COVID-19 restrictions, whereas those in Study 2.0 reported an augmentation of cognitive activities. No significant differences between Study 1.0 and 2.0 were found in the other three brain health behaviors. Figure 2 depicts the impact of the COVID-19 pandemic on the four main outcomes by diagnostic status (e.g., SCD/at risk, MCI, Dementia). However, none of the ANOVAs was significant.

**Table 2:** Independent sample, two-tailed *t*-test assuming unequal variance between intervention type (intervention [BHC] vs. control [PCE]).

Change in behaviors	BHC (*M*) *n* = 11	PCE (*M*) *n* = 13	*t*-test	*p*-value
Physical activity	−.55	−.85	−.584	.566
Diet	0	−.08	−.213	.833
Cognitive activity	−.27	0	.769	.450
Social engagement	−1.55	−1.77	−.668	.516

Note. BHC, Brain Health Champion; PCE, Physician Counseling and Education.

No significant differences between intervention type in change in physical activity, diet, cognitive activity, or social engagement were found. No effect of intervention type. Change in behavior reported on a 5-point Likert scale: 2 – Significantly More/Better; 1 – Somewhat More/Better; 0 – About the Same; −1 – Somewhat Less/Worse; −2 – Significantly Less/Better. Values are averages from survey responses.

**Table 3:** Independent sample, two-tailed *t*-test assuming unequal variance between study type (study 1.0 vs. Study 2.0).

Change in behaviors	Study 1.0 (*M*) *n* = 14	Study 2.0 (*M*) *n* = 10	*t*-test	*p*-value
Physical activity	−.79	−.60	−.330	.746
Diet	.21	−.40	1.715	.106
Cognitive activity	−.57	.50	−4.091	<.001*
Social engagement	−1.64	−1.70	.166	.871

Note. Significant difference found between Study 1.0 vs. 2.0 in self-reported change in cognitive activity levels. Change in behavior reported on a 5-point Likert scale: 2 – Significantly More/Better; 1 – Somewhat More/Better; 0 – About the Same; −1 – Somewhat Less/Worse; −2 – Significantly Less/Better. Values are averages from survey responses.

### Additional health questionnaires results

**Figure 3:**
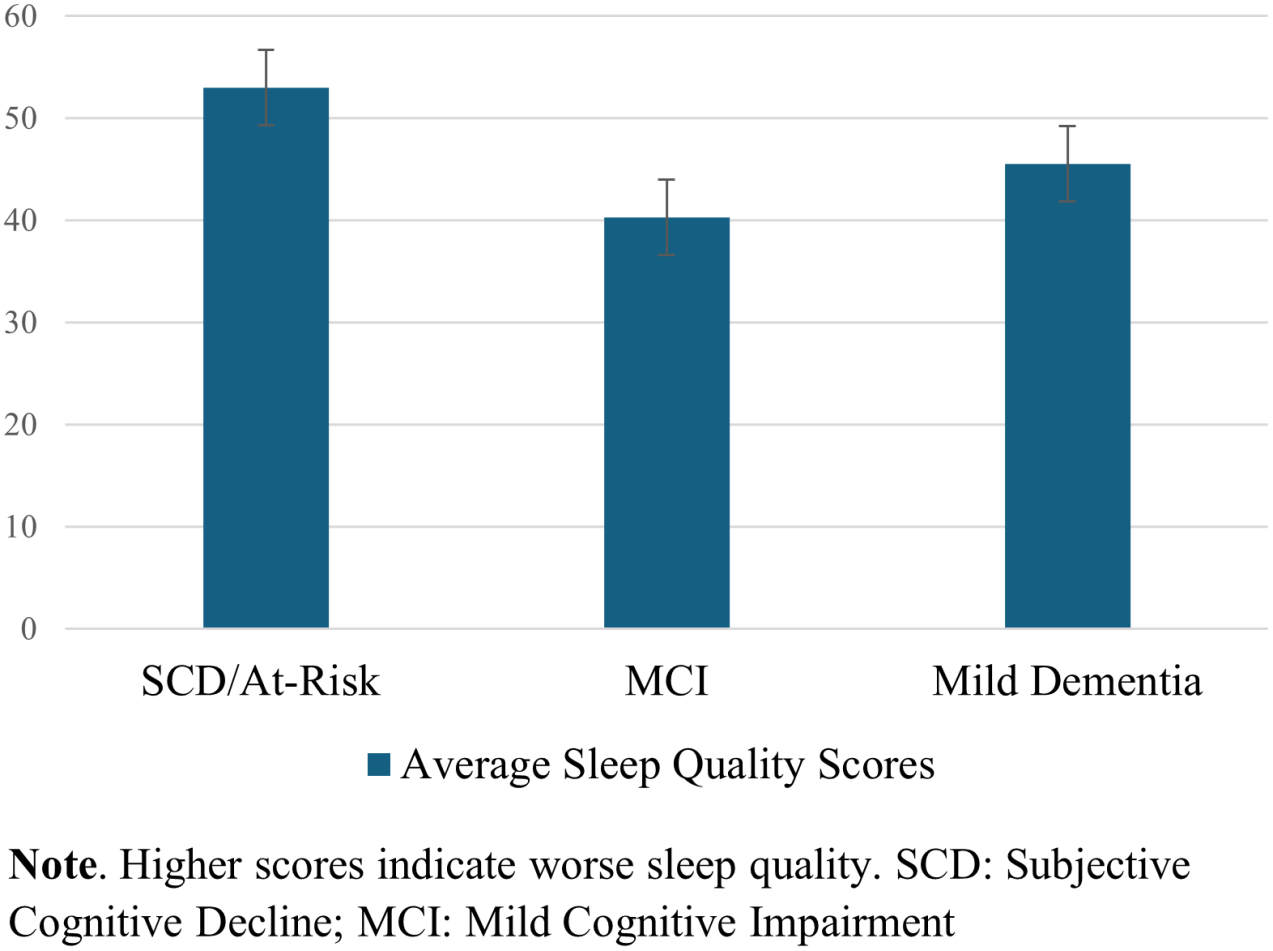
Sleep quality reports across diagnostic groups.

One-way ANOVA test of between-subject factors (diagnostic group) with *post hoc* tests (using the Bonferroni correction to adjust *p*-values) indicated that, over the surveyed month of early COVID-19 pandemic, a difference in self-reported sleep quality between at least two groups was found [*F*(2, 24) = 3.779, *p* = .04]. Specifically, worse sleep quality was endorsed in SCD/At-risk participants than in MCI participants (*p* = .036), but not significantly worse than in participants with mild dementia, regardless of intervention type. See Figure 3. The one-way ANOVAs were run for the GAD-7 (anxiety) and PHQ-9 (depression) scores, with no significant differences between diagnostic groups.

### Feedback survey results

We found that 40% of our participants, from both the intervention and active control in BHC Study 2.0, felt that their involvement in the BHC study had a positive impact on their response to additional personal stresses (e.g., feeling lonely or isolated; not being able to see loved ones or close friends in-person; having medical appointments postponed) that were brought on by the pandemic. All those participants felt that the positive impact was due to knowing that they were being encouraged by a health care team to adopt behaviors that promote health, whether the encouragement was from personalized coaching or from periodically receiving educational materials. Only 20% of participants from Study 1.0 reported that their involvement in the program had some impact on their response to additional stressors or routine disruptions during the pandemic. The rest of the 1.0 participants reported that the BHC study had no impact on their response to additional personal stressors.

Six themes were identified amongst the open-response question on the feedback survey (e.g., “Please describe any other thoughts, feelings, or personal reactions that you have about the COVID-19 pandemic and/or the Brain Health Champion Study any additional information”): personal concerns, concerns for others, optimism for future, grateful for study participation, experienced a negative behavior change, and experienced a positive behavior change. Seventy-five percent of participants answered this question. Responses could be categorized as more than one theme. Thirty-five percent of participants (*n* = 6) expressed concerns for others (i.e., frontline workers, family, friends, etc.), followed by 29% (*n* = 5) of participants who had personal concerns for their own wellbeing, 24% (*n* = 4) were grateful for their participation in the BHC study, 24% (*n* = 4) experienced a negative behavior change (e.g., reduction in social engagement), 12% (*n* = 2) were optimistic for the future, and 12% (*n* = 2) experienced a positive behavior change (e.g., improvements in diet). All participants that were grateful for their participation were a part of BHC Study 2.0. One participant wrote, “I would not have signed up for Tai Chi had I not joined the study. It brought me back to thinking about psychology and self-improvement.” Another wrote, “My physical and social activities have been substantially curtailed during this [pandemic] period, but this is not a reflection of my participation in the study. In fact, I became increasingly appreciative of my weekly chats with my BHC coach during this period.”

## Discussion

We studied the extent to which COVID-19 containment strategies during the early months of 2020 impacted the behaviors adopted by active participants or completers in our ongoing Brain Health Champion (BHC) study, an investigation examining the use of health coaches and digital technologies to promote brain healthy behaviors in older individuals with either risk factors for dementia, mild cognitive impairment, or mild dementia. Overall, among 25 participants from two different study cohorts separated by more than 1 year, we found that impacts of the pandemic on behaviors differed depending on the kind of brain healthy behavior.

### Impacts on social engagement, cognitive and physical activity

The pandemic significantly decreased self-reported social engagement and physical activity in all study participants, regardless of intervention type, study cohort (Study 1.0 vs. 2.0), and diagnosis (SCD/at-risk, MCI, or dementia). These findings suggest that neither those in the 6-month health-coaching intervention of our study compared to those getting usual clinical care augmented by educational handouts, nor those who recently completed health-coaching compared to those who completed it up to 18 months prior, were protected from the deleterious effects of public health containment strategies on maintenance of these behaviors. Pandemic restrictions and social distancing recommendations also did not spare any diagnostic category, suggesting that both cognitively normal older adults with dementia risk factors and those with cognitive impairment experienced notable decreases in social and physical activity. The effects on these behaviors might be predicted as physical activity and social engagement are more commonly achieved outside of the home than other behaviors, and pandemic restrictions largely limited activities outside the home.

On the other hand, cognitive activity levels and diet quality were relatively spared across participants, regardless of diagnosis or whether they were being coached or provided periodic counseling and educational materials. Maintaining a high quality, healthy diet and cognitive activity levels are behaviors more easily achieved inside the home than socialization and physical activity. Notably, we did find that participants from Study 2.0 reported an increase in their cognitive activity during pandemic restrictions, while participants from Study 1.0 reported a decrease. Because at the onset of containment strategies, Study 2.0 participants were all still enrolled in, or had recently completed, the intervention, it is possible they were more motivated to participate in additional cognitively stimulating activities than Study 1.0 participants, who had finished the study's intervention one year or longer before the start of the pandemic.

For participants in the active health coaching arm (BHC) of Study 2.0 at the onset of pandemic restrictions, coaches tried to adapt their weekly, personalized video sessions to encourage the adoption of new or modified activities in which to participate while at home. However, additional analysis showed no difference in cognitive activity levels between Study 2.0 participants in the coaching arm (BHC) vs. the counseling and education arm (PCE; active control). Thus, motivation to increase cognitive activity levels could have been the result of arm-independent factors, such as a recency effect of having participated in a research study in any capacity that promotes healthy behaviors and/or random differences of general motivation to participate in an active lifestyle-focused study.

Regarding factors that might have stabilized or lessened declines in physical activity and socialization, it may be that at the time of the survey, which was only two months after pandemic restrictions became widespread in the greater New England, U.S. region where the study was conducted, it was too early to see any “protective” effects of coaching adaptations for those actively enrolled. It is also possible that forced changes of routine during these early months of the COVID-19 pandemic were so substantial for participants, that no coaching effort, whether ongoing, personalized, or completed once-weekly, would have succeeded in helping them maintain brain healthy behaviors.

### Impacts on sleep

Self-reported sleep was considerably more disrupted with the onset of the pandemic for at-risk/SCD participants than MCI participants, and trends showed that at-risk/SCD participants endorsed more anxiety and depression than MCI participants. These findings are challenging to interpret, given the known higher prevalence of these neuropsychiatric symptoms in patients with MCI due to AD or due to other etiologies compared to age-matched controls (33). Maintaining structured, daily routines is one essential, non-pharmacologic approach to managing behavioral symptoms in patients with cognitive impairment and dementia. Disruption of routines commonly “un-mask” neuropsychiatric and behavioral symptoms in this population, likely because of their vulnerable brain substrate and diminished capacity to adapt to change (34). It is likely that the challenge and frustrations with these changes of routine could have yielded untoward psychological effects, with direct impact on affective functioning and sleep. Further investigation is necessary to determine whether disruption of sleep and brain-healthy behaviors in these populations improved with the ultimate lifting of the vast majority COVID-19-related restrictions.

### Digital health and clinical research during public health emergencies

Importantly, we have reported elsewhere that digitally-facilitated health coaching in the BHC study to promote brain healthy behaviors in older individuals who are either cognitively normal or have cognitive impairment is both feasible, desired, and appears to be effective (12, 35). Despite feasibility, the pandemic's restrictions and physical distancing recommendations seemed to undermine most maintenance of behavior changes achieved through the interventions, with the exception of cognitive activity levels in one cohort (Study 2.0). Notably, the negative effects of these pandemic containment strategies cut across time in our study, impacting individuals regardless of how proximal to the onset of restrictions they completed their program. One consideration here is that, like any behavior-change benefits accrued from clinical care or other external, community/family influences, the advantages derived from interventional, clinical research programs are highly susceptible to environmental influences.

As noted above, our results do suggest that having been actively enrolled in or recently completing the study which had a digital platform-augmented arm (Study 2.0) yielded some protective effects. Participants from Study 2.0 were more likely to engage in cognitive activity while participants from Study 1.0 were less likely to engage in cognitive activity during COVID-19 restrictions. Given our results, which demonstrated limits of a digital health/coaching intervention's ability to combat the environmental effects on health caused by pandemic-related restrictions, future behavioral research is needed to better understand how to improve outcomes, implementation, and possibly timing of such interventions.

### Study limitations

As mentioned above, our study has limitations. First, even while conceived of as pilot studies with aim to understand feasibility, our overall sample size is relatively small, especially within the diagnostic category of dementia, which was an independent variable in analysis. The size of the sample limits the conclusivity of our interpretations. Second, our sample is limited to predominantly White, urban or suburban-dwelling residents of New England in the U.S. Given the myriad sociodemographic and cultural factors that influence outcomes of research in general, and particularly behavior change and health, it is important to consider how these results might generalize to other U.S. or global sub-populations. Third, the pooled data from two different cohorts of study completers, some of whom completed the study one or more years prior, make results more difficult to interpret. Next, we do not have longitudinal data beyond one-year post-intervention during “normal” (non-pandemic) times to understand how well BHC study completers maintain their brain health behaviors. It may be that changes in the behavior of participants who completed the study more than one year prior may be better or equally attributed to factors separate from the pandemic, like a dwindling impact of the intervention, which we could not consider in our interpretation of results. Finally, we chose to focus on self-reported change of behavior just after the onset of pandemic restrictions, rather than current behavior at the time of the survey, and relatedly, did not quantify immediate-pre-pandemic (baseline) levels of brain healthy behaviors and neuropsychiatric symptoms in participants, except for a few who had just completed the study. More research is needed to understand how behavioral interventions, like the technology-enhanced health coaching programs in the BHC study, can help sustain behaviors at six months, one year, and beyond, especially in the face of real-world crises.

## Conclusion

Our study provides pertinent information regarding the impact of the COVID-19 pandemic's restrictions on the brain health behaviors of both cognitively normal and cognitively impaired older individuals. Our results showed that actively participating in a clinical care-embedded brain health behavior-promoting study at the time of pandemic restrictions improved cognitive activity engagement as compared to participants who completed the study one or more years prior to the pandemic. This suggests that for at least some behaviors, the study's interventions had some protective effects against the impact of COVID-19 containment strategies. Our study also provided an opportunity to understand whether there were differences in the behavior of older adults who participated in a digital health-enhanced coaching intervention vs. those who received usual care augmented by educational materials after the implementation of pandemic-related containment strategies. The results were clear that the pandemic profoundly impacted socialization and physical activity, regardless of the program individuals were randomized or their cognitive status. However, it is important to note that our study has a relatively small sample size, which may limit the generalizability of our findings. Ongoing research is needed to understand what elements and implementation aspects of digital health in clinical care might be optimal in helping older individuals maintain their brain health in the face of significant disturbances to daily life.

## Data Availability

The raw data supporting the conclusions of this article will be made available by the authors, without undue reservation.
