# Supplementary material for: Telehealth coaching in older adults, behavior change, and impacts of the COVID-19 pandemic: analyses from The Brain Health Champion Study
Source: Front Digit Health. 2025 Apr 17;7:1510804. doi: 10.3389/fdgth.2025.1510804 (PMC12043590; doi:10.3389/fdgth.2025.1510804)
Supplement: Supplementary file 1 [file Presentation1.pdf]

Questionnaire for BHC Study participants:
Response During COVID-19 Pandemic

1) Have you experienced some increased feelings of stress and anxiety during the coronavirus pandemic in the U.S./New England Area (starting ~ March 2020), compared to the period before?

    Qualify (Scale of 1-5): Significantly more stress/anxiety, A little more stress/anxiety, About the same as before, A little less stress/anxiety, Significantly less stress/anxiety

2) Have you been impacted by COVID-19 (the coronavirus) by either becoming infected yourself, by someone in your immediate family/extended family or a friend who was infected, or by living with someone who has gotten infected?

    Yes/No

    If Yes:
    A) [Check all that apply]
        a. I got sick with COVID-19
        b. A close contact of mine got sick (e.g., family member, friend, or other acquaintance you see regularly)
        c. Someone living in my home got sick

    B) If either you have been infected or someone who you were in direct/close contact with got infected, were you told to **self-isolate** or **quarantine (for 2 weeks or longer)** by the health care system, your doctor, or government/public health recommendations, *or*, did you do so on your own? Yes/No
        If yes, choose: self-isolate; quarantine;
        If yes, choose: healthcare/government/press; self

3) Have you been practicing "social distancing", regardless of any direct experience or contact with someone infected? This means that you have significantly limited your outside-the-home social interactions, cancelled travel plans, and kept physically apart from anyone you see face-to-face (e.g. cashier at the grocery store; gas station attendant; neighbors)

    Yes/No

    If Yes:
    A) How long have you practiced social distancing at the time you are responding to this questionnaire? (Write-in);

    B) Characterize to what extent you have been able to maintain this practice:
        1. Very strictly – no face-to-face contact with anyone except those living in my household

2. Fairly strictly – limited face-to-face contact with those outside of my household (1-3 times per week); (e.g., talking with cashier while buying essential items, interacting with extended family/community members in very small groups

3. Reasonably strictly – some face-to-face contact with those outside my household (4 or more times per week, or daily)

C. [Check all the apply] Which of these have you experienced related to social distancing?

1. Boredom and frustration
2. Loneliness
3. Worsening of prior feelings of social isolation
4. New feelings of social isolation
5. Worsening of prior feelings of depression or anxiety
6. New feelings of depression or anxiety
7. Lessened fear related to getting the infection
8. Feelings of benevolence of doing your own part to slow the spread of disease
9. A greater feeling of connectedness to society/friends/workmates, as we are all contributing to a common goal
10. Other (please explain)

D. [Check all that apply] If you are not practicing social distancing, what are your reasons for not doing so?

1. It is difficult/impossible to do this in my current living situation and/or with my work/caregiving obligations
2. It would be detrimental to my own mental health
3. I am not sure how much I can personally help contain the virus
4. I question whether the social distancing guidelines are too strict and maybe not necessary
5. Other (please explain)

E. How does your current level of physical activity compare to your activity prior to March 2020?

Significantly less; Somewhat less; About the same; Somewhat more; Significantly more

a. Please explain how your physical activity has or has not changed due to the pandemic. (Write-in)

F. How does your current diet compare to your diet prior to March 2020?

Significantly worse; Somewhat worse; About the same; Somewhat better; Significantly better

a. Please explain how your diet has or has not changed due to the pandemic. (Write-in)

G. How does your current level of cognitive activity compare to your activity prior to March 2020?

Significantly less; Somewhat less; About the same; Somewhat more; Significantly more

a. Please explain how your cognitive activity has or has not changed due to the pandemic. (Write-in)

H. How does your current level of social engagement compare to your engagement prior to March 2020?

Significantly less; Somewhat less; About the same; Somewhat more; Significantly more

a. Please explain how your social engagement has or has not changed due to the pandemic. (Write-in)

I. What social interactions, activities, or hobbies of yours have been disrupted or cancelled during this pandemic? (Open-ended; write-in answers)

4) Have you felt lonely much of the time in the past week?
Yes/No

5) Do you think your involvement in the Brain Health Champion study has had any impact on your response to additional personal stresses (e.g. feeling lonely or isolated; not being able to see loved ones or close friends in-person; having medical appointments postponed) that were brought on by the pandemic?

Yes/No

If Yes:
A) Do you think the study involvement has had an overall positive impact or negative impact? Please describe. (Write-in)
B) What aspects of the study have had an impact, if any, on your coping response to the pandemic and/or your quality of life during this time? (Check all that apply)

1. Knowing you are being encouraged by a health care team to adopt behaviors that promote health

2. Knowing that you are connected through a weekly video call and possible text messaging to the health coach (**for BHC arm only**)
3. Knowing that you are involved in a medical research study of any kind
4. Knowing that you are involved in a medical research study that is specifically trying to advance our understanding of the brain and wellness
5. Your relationship with your health coach in this study (**for BHC arm only**)
6. The educational materials about brain health that have been distributed to you as part of the study

6) How many people live in your home (including yourself)? (Write-in)

7) **(BHC arm only):** How much of a comfort and/or reassurance for you has it been to be able to check in regularly with brief video interactions or text messaging with someone from the study during this time?

Significant comfort; Some comfort; Neutral; Some Discomfort; Significant discomfort

(PCE arm only): Do you think it would give you additional comfort and/or reassurance for you to be able to check in regularly with brief video interactions or text messaging with someone from the study during this time?

Significant comfort; Some comfort; Neutral; Some Discomfort; Significant discomfort

8) On average, about how many people per week did you see or spend time with in-person *prior* to March 2020, when the U.S./local governments started recommending social distancing? (answer)
   a) 0-1 person
   b) 2-3 people
   c) 4-5 people
   d) 6-7 people
   e) 8 or more people

9) On average, about how many people per week do you see or spend time with in-person now, during the social distancing recommendations by U.S./local government? (answer)

   a) 0-1 person
   b) 2-3 people
   c) 4-5 people
   d) 6-7 people
   e) 8 or more people

10) On average, about how many people per week do you connect to during this crisis either by phone or video calling? (answer)

11) Please describe any other thoughts, feelings, or personal reactions that you have about the COVID-19 pandemic and/or the Brain Health Champion Study.

12) Is there other information that you would like to share?
